# Association between awareness of nutrition labeling and high-density lipoprotein cholesterol concentration in cancer survivors and the general population: The Korean National Health and Nutrition Examination Survey (KNHANES) 2010–2016

**DOI:** 10.1186/s12885-018-5196-6

**Published:** 2019-01-07

**Authors:** Ji Sung Yoo, Kyu-Tae Han, Seung Hyun Chung, Eun-Cheol Park

**Affiliations:** 1 Department of Rehabilitation Medicine, Research Institute and Hospital, National Cancer Center, Goyang, Republic of Korea; 2 Division of Cancer Management Policy, National Cancer Control Institute, National Cancer Center, Goyang, Republic of Korea; 3 Institute of Health Services Research, Yonsei University College of Medicine, Seoul, Republic of Korea; 4 Department of Preventive Medicine, Yonsei University College of Medicine, Seoul, Republic of Korea

**Keywords:** Nutrition facts, HDL-C, Cancer survivors, Tertiary cancer prevention, Nutrition labeling

## Abstract

**Background:**

Nutrition labeling has been found to affect the amount and type of food intake, with certain groups in the population, such as cancer survivors, being more aware of this information. A higher awareness of nutrition labeling is inversely related to the risk of dyslipidemia. This study therefore assessed the association between awareness of nutrition labeling and high-density lipoprotein cholesterol (HDL-C) concentration among cancer survivors in South Korea and in the general population of subjects without a history of cancer.

**Methods:**

This cross-sectional analysis evaluated 25,156 adults who participated in the Korean National Health and Nutrition Examination Surveys (KNHANES) from 2010 to 2016. Factors influencing the association between awareness of nutrition labeling and HDL-C concentration in cancer survivors and the general population were determined by multiple regression analysis.

**Results:**

Of the 25,156 participants, 2.88% were cancer survivors and 97.12% had no history of cancer. HDL-C concentrations were higher in subjects who were aware of nutrition labeling than in subjects who were not. Checking or using nutrition labeling had a greater effect on the management of HDL-C concentration for cancer survivors than for the general population.

**Conclusion:**

Awareness of nutrition labeling was associated with better outcomes, including higher controlled HDL-C levels, and reductions in factors increasing the risk of coronary artery disease and cancer, especially in cancer survivors. Health policymakers or medical professionals should develop programs to promote the use of nutrition labeling among cancer survivors in South Korea.

**Electronic supplementary material:**

The online version of this article (10.1186/s12885-018-5196-6) contains supplementary material, which is available to authorized users.

## Background

In South Korea, more than 200,000 patients were newly diagnosed with cancer in 2014. Improvements in early detection and treatment has increased the 5-year survival rate for all cancers, from 41.2% for patients diagnosed between 1993 and 1995 to 70.3% for those diagnosed between 2010 and 2014 [1]. Cancer survivors have a greater risk for cardiovascular disease, cancer recurrence, and second primary malignancies [2, 3]. Therefore, cancer survivors should be more invested in maintaining ideal body weight, a healthy diet, and a physical activity level to prevent chronic diseases, cancer recurrence, and second primary cancers [4]. Dietary interventions have been shown to improve diet quality, body weight, and nutrition-related biomarkers [4]. Low-fat diets have been found to promote changes in serum lipids, cytokines, and angiogenic factors [5]. Plasma concentrations of high-density lipoprotein-cholesterol (HDL-C) have shown a strong inverse relationship to the risk of developing coronary artery disease (CAD) and cancer [6–8]. On the other hand, a low concentration of HDL-C is associated with obesity [7].

Nutrition labeling of foods became compulsory in South Korea in 1995 [9], to assist consumers in making reasonable choices based on nutrition values by confirming the nutritional properties of processed foods [9, 10]. Nutrition labeling has been found to affect the intake of total fats, carbohydrates and saturated fats, as well as awareness of the nutritional contents of foods, which may be helpful in managing certain chronic diseases [11]. Moreover, greater awareness of nutrition labeling in a South Korean population was inversely related to the risk of dyslipidemia, especially regarding imbalances of HDL-C and triglycerides [12]. The introduction of nutrition labeling of foods in South Korea was expected to provide food-related health information to the South Korean population, cancer survivors included, to help them better manage their health. For that reason, this comparative study aimed to assess the association between individual nutrition concerns and HDL-C concentration among South Korean cancer survivors and the general population without a history of cancer.

## Methods

### Study population

The data used in this study were obtained from the Korea National Health and Nutrition Examination Surveys (KNHANES) V–VII, 2010–2016. KNHANES are cross-sectional surveys conducted annually since 1998 by the Korea Centers for Disease Control (KCDC) using a stratified, multistage, cluster sampling design. Each survey consists of three types of questionnaire: A Health Interview Survey, a Health Examination Survey, and a Nutrition Survey. Of the 71,723 respondents, those diagnosed with cancer and undergoing cancer treatment or respondents without blood test results were excluded. In addition, to compare the impact of nutrition information awareness between cancer survivors and the general population, we only included persons aged over 30 years. We also excluded those who did not respond to the nutrition information awareness questions in order to reduce the uncertainty caused by incomplete surveys. A total of 25,156 participants were deemed eligible for this study.

### Variables

To compare differences between cancer survivors and the general population in their awareness of nutrition information, the main outcome variable was defined as serum HDL-C concentration on the survey date. A 40–60 mg/dL concentration of HDL-C was considered normal.

Awareness of nutrition information was divided into three categories: 1) checks nutrition information and makes label-dependent purchase decisions; 2) checks nutrition information but does not make label-dependent purchase decisions or is aware of nutrition information but does not check them when making food purchase decisions; and 3) is unaware of nutrition information. Other independent variables included cancer survivor, sex, age, level of education, household income, body mass index (BMI), aerobic exercise habits, smoking status, high-risk drinking, any family history of hyperlipidemia, survey year, stress awareness, subjective health status, frequency of eating-out, total cholesterol concentration, triglyceride concentration, low-density lipoprotein-cholesterol (LDL-C), and average daily intake (energy/carbohydrates/fats).

Cancer survivors were defined as cancer patients who were not undergoing cancer treatment at observation time, with other subjects defined as the general population. Age categories included age < 30 years, 30–39 years, 40–49 years, 50–59 years, and ≥ 60 years. BMI categories included BMI < 23 kg/m^2^ (underweight or normal), BMI 23–25 kg/m^2^ (overweight), and BMI > 25 kg/m^2^ (obese). A family history of hyperlipidemia was defined diagnostically in first-degree relatives. The cutoff for weekly aerobic exercise time was defined as 150 min. High-risk drinking was defined as the consumption of more than seven (males) or five (females) drinks on a single occasion at least twice a week. The average daily intake was based on the food intake questionnaire, which was designed as an open-ended survey for reporting various dishes and foods using the 24-h recall method [13]; energy intake was calculated using these data.

### Statistical analysis

Categorical variables were reported as frequency and percentage, and continuous variables as mean and standard deviation. Chi-square tests were performed to determine the association of independent variables and awareness of nutrition labeling for both cancer survivors and the general population, as well as to determine the relationship of each variable with cancer status. The relationship of continuous variables with HDL-C concentrations was determined by analysis of variance (ANOVA) and is displayed in Additional file 1: Table S1. After adjusting for covariates in cancer survivors and the general population, multiple linear regression analysis, utilizing gamma distribution, was performed to assess the relationship between the use of nutrition data and HDL-C concentration. Subgroup analyses were also performed by sex, frequency of eating-out, and subjective health status. Sampling weight was applied to each participant to generalize the data. All statistical analyses were performed using SAS version 9.4 (Cary, NC, USA).

## Results

Of the 25,156 subjects, 724 (2.88%) were cancer survivors and 24,432 (97.12%) had no history of cancer. Table 1 shows the characteristics of both groups. We found that approximately 20% of all respondents actively checked and made purchase decisions based on nutrition information, but that the percentage was significantly lower in cancer survivors than in the general population (16.30% vs. 19.32%, *p* < 0.0001). Cancer survivors were significantly older and of lower socioeconomic status but had healthier lifestyles than the general population in their behaviors of smoking, drinking, eating-out habits, and daily nutritional intake.

**Table 1:** General characteristics of the study population

Variables	Cancer Survivor		General Population		*P*-value
	N	%	N	%	
Awareness on nutrition labelling					
Checks nutrition facts and makes labeling-dependent purchase decisions	107	16.30	4468	19.32	<.0001
Checks nutrition facts but does not make labeling-dependent purchase decisions/ Aware of nutrition facts but does not check them when making food purchase decisions	271	40.84	11,681	52.02	
Unaware of nutrition facts	346	42.87	8283	28.66	
Sex					
Male	281	39.88	10,131	46.84	0.1530
Female	443	60.12	14,301	53.16	
Age (years)					
30–39	26	5.11	5679	27.64	<.0001
40–49	86	16.00	5468	27.82	
50–59	150	23.28	5047	21.82	
60+	462	55.61	8238	22.72	
Educational level					
Under high school graduation	562	75.11	15,778	60.63	<.0001
Bachelor’s degree	137	20.52	7550	34.32	
Master’s degree or above	25	4.37	1104	5.04	
Household income					
Low	238	31.09	4796	15.71	<.0001
Mid-low	169	22.85	6126	25.55	
Mid-high	156	21.83	6697	29.41	
High	161	24.22	6813	29.34	
BMI					
< 23	357	51.37	10,798	42.86	0.0239
23–25	158	19.96	5816	24.00	
> 25	209	28.68	7818	33.14	
Aerobic exercise habits					
Yes	198	28.44	7048	31.46	0.3800
No	526	71.56	17,384	68.55	
Smoking status					
Non-smoker	652	89.51	19,871	77.06	<.0001
Smoker	72	10.49	4561	22.94	
High-risk drinking					
No	689	93.83	22,032	87.69	<.0001
Yes	35	6.17	2400	12.31	
Family history for hyperlipidemia					
No	704	96.60	23,269	94.99	0.0123
Yes	20	3.40	1163	5.01	
Survey year					
2010	104	12.80	4089	14.77	0.0484
2011	104	13.53	3983	15.17	
2012	102	12.84	3637	14.54	
2013	100	16.38	3312	14.17	
2014	88	13.00	3121	13.64	
2015	106	14.10	2969	13.68	
2016	120	17.35	3321	14.03	
Stress awareness					
Low	559	75.90	18,566	74.82	0.4488
High	165	24.10	5866	25.18	
Subjective health status					
Good	163	21.56	7965	33.15	<.0001
Normal	338	47.32	12,123	50.57	
Bad	223	31.12	4344	16.28	
The frequency of eating out					
More than five times a week	118	17.24	7895	38.29	<.0001
Less than four times a week	606	82.76	16,537	61.71	
Average amount of total energy intake (Kcal)	1754.49	±29.16	2057.86	±8.27	<.0001
Average amount of daily carbohydrate intake (g)	297.97	±4.99	319.75	±1.22	0.0030
Average amount of daily fat intake (g)	31.42	±1.09	43.20	±0.32	<.0001
Total cholesterol (mg/dL)	190.02	±1.50	192.54	±0.30	0.3827
Triglyceride (mg/dL)	134.51	±5.94	138.05	±1.02	0.3745
LDL cholesterol(mg/dL)	112.67	±1.43	114.80	±0.28	0.7387
Total	724	100.00	24,432	100.00	

*BMI* body mass index, *LDL* low-density lipoprotein

Based on previous reports, nutrition factors could have a positive effect on the management of triglyceride and HDL-C [14]. Among four key indicators: total cholesterol, triglyceride, HDL-C, and LDL-C, the triglyceride and HDL-C concentrations were associated with active use of nutrition information. Similarly positive impacts of nutrition labelling were reported in a previous study, showing a decrease of triglycerides and an increase of HDL-C by active use of nutrition information [12]. In this study, we focused on comparing the positive impacts of nutrition information awareness between cancer survivors and the general population. However, triglyceride results alone were not meaningful among cancer survivors (Additional file 1: Table S2). Thus, we decided to only focus on HDL-C to compare the positive impact of using nutrition information. Table 2 shows the findings of multiple gamma linear regression analysis for the relationship between nutrition information awareness and HDL-C level in all respondents. HDL-C concentrations were higher in subjects who checked, or were aware of, nutrition information (Relative Risk [RR]: 1.0017, 95% Confidence Interval [CI]: 1.0004–1.0031, *p* = 0.0131), and while there was no significant difference in these subjects, those who made purchase decisions based on nutrition labelling had positive increases of HDL-C (*p* = 0.1785).

**Table 2:** Multiple regression analysis of the association between nutrition labeling awareness and subject characteristics

Variables	RR	95% CI		*P*-value
Awareness on nutrition labelling				
Checks nutrition facts and makes labeling-dependent purchase decisions	1.0012	0.9995	1.0030	0.1785
Checks nutrition facts but does not make labeling-dependent purchase decisions/ Aware of nutrition facts but does not check them when making food purchase decisions	1.0017	1.0004	1.0031	0.0131
Unaware of nutrition facts	1.0000	–	–	–
Cancer				
Cancer Survivor	0.9985	0.9950	1.0020	0.3928
General population	1.0000	–	–	–
Sex				
Male	0.9965	0.9951	0.9978	<.0001
Female	1.0000	–	–	–
Age (years)				
30–39	1.0032	1.0013	1.0051	0.0010
40–49	1.0036	1.0018	1.0053	<.0001
50–59	1.0026	1.0009	1.0042	0.0024
60+	1.0000	–	–	–
Educational level				
Under high school graduation	1.0005	0.9980	1.0029	0.7066
Bachelor’s degree	1.0014	0.9991	1.0038	0.2356
Master’s degree or above	1.0000	–	–	–
Household income				
Low	0.9993	0.9975	1.0011	0.4727
Mid-low	1.0012	0.9998	1.0026	0.0927
Mid-high	1.0012	0.9999	1.0025	0.0694
High	1.0000	–	–	–
BMI				
< 23	1.0019	1.0007	1.0032	0.0024
23–25	1.0020	1.0007	1.0034	0.0035
> 25	1.0000	–	–	–
Aerobic exercise habits				
Yes	1.0000	–	–	–
No	1.0002	0.9990	1.0013	0.7728
Smoking status				
Non-smoker	1.0000	–	–	–
Smoker	0.9956	0.9942	0.9970	<.0001
High-risk drinking				
No	1.0000	–	–	–
Yes	1.0047	1.0030	1.0065	<.0001
Family history for hyperlipidemia				
No	1.0000	–	–	–
Yes	0.9978	0.9955	1.0001	0.0604
Survey year	0.9993	0.9991	0.9996	<.0001
Stress awareness				
Low	1.0000	–	–	–
High	0.9998	0.9987	1.0010	0.7951
Subjective health status				
Good	1.0001	0.9985	1.0017	0.8931
Normal	0.9997	0.9983	1.0012	0.7262
Bad	1.0000	–	–	–
The frequency of eating out				
More than five times a week	1.0000	–	–	–
Less than four times a week	1.0017	1.0005	1.0029	0.0060
Average amount of total energy intake (per 100 Kcal)	1.0004	1.0003	1.0006	<.0001
Average amount of daily carbohydrate intake (per 10 g)	0.9998	0.9997	0.9999	<.0001
Average amount of daily fat intake (per 10 g)	0.9995	0.9992	0.9998	0.0008
Total cholesterol (per 10 mg/dL)	1.2096	1.2090	1.2102	<.0001
Triglyceride (per 10 mg/dL)	0.9620	0.9619	0.9621	<.0001
LDL cholesterol(mg/dL)	0.9813	0.9813	0.9814	<.0001

*BMI* body mass index, *LDL* low-density lipoprotein

HDL-C concentrations did not differ between cancer survivors and the general population. By other covariate analyses, female or younger subjects had higher HDL-C level than male or older subjects. Additionally, subjects with lower BMI were more likely to have higher HDL-C concentrations than the obese group. Subjects with heathy behaviors: non-smoker, mild drinker, or people with less frequency of eating-out, had healthier HDL-C concentrations. The results of the daily intake of nutrition survey show that respondents with lower carbohydrate or fat intake had higher HDL-C. In addition, triglyceride and LDL-C concentrations were inversely related to HDL-C concentration.

Table 3 displays the results of multiple linear regression analysis for cancer survivors and the general population. The active use of nutrition information had a positive correlation with higher HDL levels, particularly in cancer survivors. When compared to cancer survivors who did not check nutrition information, significantly higher HDL-C concentrations were seen in cancer survivors who checked and made label-dependent purchase decisions (RR: 1.0117, 95% CI: 1.0001–1.0233, *p* = 0.0479), as well as those who checked or were aware of nutrition information, but did not make labeling-dependent purchase decisions (RR: 1.0121, 95% CI: 1.0036–1.0205, *p* = 0.0050). In the general population, only those who checked or were aware of nutrition information had significantly higher HDL-C concentrations compared to those unaware of nutrition information (RR: 1.0015, 95% CI: 1.0001–1.0028, *p* = 0.0349). Covariate analyses showed that female, younger, or low BMI was associated with higher HDL-C in only the general population, and not cancer survivors. Similar results were observed among those with unhealthy behaviors such as smoking, drinking, or eating-out, which were only significant in the general population. However, daily carbohydrate or fat intake was inversely associated with HDL-C concentrations in both groups, and triglyceride and LDL-C concentrations were inversely related to HDL-C concentration.

**Table 3:** Multiple regression analysis of the association between nutrition labeling awareness and outcome variables in cancer survivors and general population

Variables	Cancer Survivor				General Population			
	RR	95% CI		*P*-value	RR	95% CI		*P*-value
Awareness on nutrition labelling								
Checks nutrition facts and makes labeling-dependent purchase decisions	1.0117	1.0001	1.0233	0.0479	1.0009	0.9992	1.0027	0.3042
Checks nutrition facts but does not make labeling-dependent purchase decisions/ Aware of nutrition facts but does not check them when making food purchase decisions	1.0121	1.0036	1.0205	0.0050	1.0015	1.0001	1.0028	0.0349
Unaware of nutrition facts	1.0000	–	–	–	1.0000	–	–	–
Sex								
Male	1.0034	0.9951	1.0118	0.4217	0.9964	0.9950	0.9978	<.0001
Female	1.0000	–	–	–	1.0000	–	–	–
Age (years)								
30–39	1.0142	0.9965	1.0321	0.1164	1.0032	1.0013	1.0051	0.0010
40–49	1.0006	0.9894	1.0119	0.9190	1.0036	1.0018	1.0054	<.0001
50–59	1.0010	0.9915	1.0106	0.8353	1.0026	1.0009	1.0042	0.0031
60+	1.0000	–	–	–	1.0000	–	–	–
Educational level								
Under high school graduation	1.0014	0.9841	1.0191	0.8719	1.0005	0.9980	1.0029	0.7201
Bachelor’s degree	1.0041	0.9866	1.0220	0.6466	1.0014	0.9990	1.0038	0.2565
Master’s degree or above	1.0000	–	–	–	1.0000	–	–	–
Household income								
Low	1.0102	0.9994	1.0209	0.0632	0.9991	0.9972	1.0009	0.3281
Mid-low	1.0110	1.0007	1.0212	0.0357	1.0010	0.9996	1.0025	0.1497
Mid-high	1.0032	0.9932	1.0132	0.5350	1.0012	0.9999	1.0025	0.0799
High	1.0000	–	–	–	1.0000	–	–	–
BMI								
< 23	1.0000	0.9918	1.0082	0.9994	1.0019	1.0007	1.0032	0.0028
23–25	0.9907	0.9813	1.0002	0.0561	1.0022	1.0008	1.0036	0.0016
> 25	1.0000	–	–	–	1.0000	–	–	–
Aerobic exercise habits								
Yes	1.0000	–	–	–	1.0000	–	–	–
No	0.9955	0.9878	1.0032	0.2527	1.0002	0.9991	1.0014	0.6806
Smoking status								
Non-smoker	1.0000	–	–	–	1.0000	–	–	–
Smoker	1.0005	0.9889	1.0122	0.9365	0.9956	0.9942	0.9970	<.0001
High-risk drinking								
No	1.0000	–	–	–	1.0000	–	–	–
Yes	1.0129	0.9965	1.0295	0.1229	1.0046	1.0029	1.0064	<.0001
Family history for hyperlipidemia								
No	1.0000	–	–	–	1.0000	–	–	–
Yes	1.0042	0.9858	1.0230	0.6550	0.9977	0.9954	1.0001	0.0560
Survey year	0.9979	0.9962	0.9996	0.0162	0.9994	0.9991	0.9996	<.0001
Stress awareness								
Low	1.0000	–	–	–	1.0000	–	–	–
High	1.0076	0.9995	1.0159	0.0675	0.9997	0.9985	1.0009	0.6400
Subjective health status								
Good	0.9987	0.9890	1.0085	0.7942	1.0001	0.9985	1.0018	0.8834
Normal	1.0010	0.9930	1.0089	0.8100	0.9997	0.9982	1.0012	0.7123
Bad	1.0000	–	–	–	1.0000	–	–	–
The frequency of eating out								
More than five times a week	1.0000	–	–	–	1.0000	–	–	–
Less than four times a week	1.0020	0.9927	1.0115	0.6676	1.0017	1.0005	1.0028	0.0069
Average amount of total energy intake (per 100 Kcal)	1.0001	0.9984	1.0017	0.9452	1.0005	1.0003	1.0006	<.0001
Average amount of daily carbohydrate intake (per 10 g)	0.9998	0.9990	1.0006	0.5958	0.9998	0.9997	0.9999	<.0001
Average amount of daily fat intake (per 10 g)	0.9991	0.9964	1.0018	0.4915	0.9995	0.9992	0.9998	0.0009
Total cholesterol (per 10 mg/dL)	1.2092	1.2057	1.2128	<.0001	1.2096	1.2090	1.2102	<.0001
Triglyceride (per 10 mg/dL)	0.9618	0.9613	0.9624	<.0001	0.9620	0.9619	0.9621	<.0001
LDL cholesterol(mg/dL)	0.9815	0.9812	0.9818	<.0001	0.9813	0.9812	0.9814	<.0001

*BMI* body mass index, *LDL* low-density lipoprotein

We performed subgroup analysis to identify factors that influence the association between awareness of nutrition information and HDL-C levels. Based on descriptive statistics for subgroups, linear differences of HDL-C levels were observed in females regardless of their group type. In the general population, females with less eating-out behavior had more linear trends than the cancer survivors. On the other hand, when compared to others, cancer survivors with good subjective health had more associations (Table 4). The results of subgroup analysis for multiple linear regression show that female gender and a reduced frequency of eating-out were significantly related to this association, regardless of cancer status. However, the impact of nutrition labelling awareness was greater in cancer survivors than the general population. In contrast, subjective health status had slightly different effects in cancer survivors and the general population (Additional file 1: Figure S1).

**Table 4:** The means and SD of HDL-C levels according to sex, frequency of eating out, and subjective health status

Subgroup	Awareness on nutrition labelling	Cancer Survivor		General Population	
		Mean	SD	Mean	SD
Sex					
Male	Active use	46.05	±10.86	46.50	±10.56
	Use	46.68	±11.04	47.06	±11.17
	None	48.95	±14.33	46.35	±11.67
Female	Active use	53.98	±12.30	55.13	±12.51
	Use	52.36	±12.14	53.76	±12.16
	None	50.39	±13.67	49.32	±11.69
The frequency of eating out					
Less than four times a week	Active use	50.74	±12.15	53.97	±12.61
	Use	51.10	±11.99	51.33	±12.39
	None	49.39	±14.10	47.82	±11.80
More than five times a week	Active use	58.08	±11.84	52.00	±12.55
	Use	47.37	±11.83	49.93	±11.86
	None	52.72	±12.60	47.93	±11.66
Subjective health status					
Good	Active use	54.15	±14.46	54.20	±12.83
	Use	48.30	±11.99	51.67	±12.23
	None	45.49	±10.66	47.94	±11.42
Normal	Active use	52.59	±12.68	53.24	±12.42
	Use	51.69	±11.61	50.48	±12.16
	None	51.27	±14.82	47.92	±11.93
Bad	Active use	49.43	±8.48	50.92	±12.51
	Use	49.24	±12.77	49.50	±12.07
	None	49.84	±14.03	47.58	±11.86

## Discussion

Healthy lifestyles, including physically activity, a normal body weight, and a healthy diet, have been associated with a reduced risk of primary cancer [15, 16]. Such lifestyles have been shown to prevent tumor recurrence, second primary cancers, and other chronic diseases in cancer survivors [4]. Therefore, many cancer survivors seek information on healthy food, dietary supplement use, complementary nutrition products, and physical activity to improve their response to cancer treatment, achieve a rapid recovery, reduce the risk of cancer recurrence, and have a good quality of life [4, 17]. In addition, improvements in dietary behavior may also reduce the adverse effects of cancer and its treatment [3, 4]. The American Cancer Society (ACS) and the World Cancer Research Fund/American Institute for Cancer Research (WCF/AICR) proposed recommendations for diet to further emphasize the importance of weight management and nutrition. These recommendations encourage the consumption of fruits, vegetables, and unrefined whole grains, and limit the intake of energy dense foods such as sugars, fats, and a variety of processed foods [3, 4, 16]. In South Korea, nutrition labeling of food products reveals nutrition information such as serving size, calories, and carbohydrate, protein, fat, sodium, sugar, cholesterol, saturated fatty acid, trans fatty acid, and unsaturated fatty acid content. Awareness of nutrition labeling has been associated with positive results on lipid profiles, especially on HDL-C and triglyceride concentrations in the South Korean population [12]. A low serum concentration of HDL-C is a risk factor for cardiovascular disease [6, 18]. Moreover, each 1 mg/dL increase in HDL-C levels reduces the risk of coronary artery disease by 2 to 3%, independent of low-density lipoprotein (LDL) and triglycerides levels [19]. Furthermore, HDL-C concentration is inversely associated with cancer incidence, regardless of sex, age, smoking status, LDL-C, BMI, and diabetes [8, 20, 21]. We therefore hypothesized that nutrition label awareness would significantly affect diet-related health status, especially HDL-C in cancer survivors, and explored the possible association between awareness and HDL-C.

This study showed that HDL-C levels were higher in respondents who checked or were aware of nutrition information than in those who did not, but the HDL-C levels did not differ between respondents who checked nutrition information and made purchase decisions based on this information. Subgroup analysis showed a similar pattern in both cancer survivors and the general population when aware of nutrition labelling and checking it. In cancer survivors, HDL-C concentrations were significantly higher in those who check nutrition facts and make labeling-dependent purchase decisions, than in cancer survivors unaware of nutrition information. This pattern was not observed in the general population. Similarly, a previous study showed that nutrition awareness, i.e., checking nutrition information and actively using it, had positive effects on outcome indicators such as HDL-C and triglyceride levels in a South Korean population that included cancer survivors [12].

This study also shows that awareness of nutrition labeling (checking) or healthy behavior groups such as low BMI, non-smoking status, less eating-out, and low daily carbohydrate/ fat intake measured in grams, were independently associated with higher HDL-C levels in the general population. High awareness of nutrition labelling may lead to improved nutritional intake, including low-fat and low-carbohydrate diets. On other hand, among cancer survivors, there were no significant associations of healthy behaviors. The appearance of these results may be caused by their clinical condition following cancer treatment as compared to the general population. Thus, they might not be as responsive to healthy behaviors as the general population. Our finding that aerobic exercise habits were not independently associated with HDL-C levels in cancer survivors, suggests that a better diet may be a more important factor for increasing HDL-C levels in South Korea. Similarly, greater adherence to ACS guidelines was related to higher social functioning scores, suggesting that diet may be an important indicator in quality of life among Korean breast cancer survivors [22].

To the best of our knowledge, this is the first study to describe the association between awareness of nutrition labeling and HDL-C level among cancer survivors in a general population in South Korea. Our use of nationwide sampling data over a 5-year period may help establish long-term health policy at the national level [12]. However, this study had some limitations. First, KNHANES collected information, including cancer history and awareness of nutrition labeling, using self-reported questionnaires, but did not collect information about cancer stage or phase of care. Thus, there may have been recall bias, which may have influenced our outcomes of interest. Second, KNHANES is conducted for the general Korean people and the survey takes 1.5 to 2 h to complete. Therefore, the number of cancer survivors was relatively small, and only healthy cancer survivors might have been able to participate. Third, nutrition label awareness could be a proxy variable for health awareness or other healthy behavior variable. Therefore, self-reported nutrition label awareness may not fully reflect their actual behavior, e.g., reading nutrition label once out of every 100 opportunities.

This study showed that high awareness of nutrition labelling is associated with high HDL-C level, which is associated with reduced risks of cardiovascular disease and/or cancer. Moreover, this association was greater in cancer survivors than in the general population. The concerns of healthy behaviors by cancer survivors the need for guidelines directed to South Korean cancer survivors are increasing. Health policy makers and medical professionals may be aided by the results of this study in developing effective measures such as body weight control, smoking cessation, reducing and maintaining a low daily fat intake as well as promoting the use of nutrition labeling across this population. Further research is needed to determine the methods of increasing individual interest in nutrition. Moreover, randomized controlled trials are needed to show the benefits of a healthy diet on HDL-C levels in cancer survivors.

## Conclusions

Awareness of nutrition labeling increased serum concentrations of HDL-C, a factor associated with coronary artery disease and the risk of cancer incidence. Active use of nutrition information correlated positively with higher HDL-C levels, especially among cancer survivors. Our findings suggest that health policy in South Korea should include the promotion of nutrition labeling for cancer survivors.

## Additional file

Additional file 1: **Table S1.** Associations between subject characteristics and serum HDL-C concentrations in cancer survivors and the general population. **Table S2.** Multiple regression analysis of the association between nutrition labeling awareness and outcome variables in cancer. **Figure S1.** The results of the subgroup analyses of the relationship between nutritional information awareness and HDL-C levels according to sex, frequency of eating out, and subjective health status. *The RR as marked to the square points was calculated by multiple regression analysis adopting gamma distribution to investigate the association between awareness on nutrition labelling and HDL-C. Results were considered statistically significant if each bar marked to SD did not reach the cut-off line of 1.0000. † The means and SD of each group were shown in Additional file 1 Figure S1. (DOCX 251 kb)

## Abbreviations

ACS: American Cancer Society
BMI: Body mass index
CAD: Coronary artery disease
HDL-C: high-density lipoprotein cholesterol
KCDC: Korea Centers for Disease Control
KNHANES: Korean National Health and Nutrition Examination Surveys ()
LDL-C: Low-density lipoprotein cholesterol
WCF/AICR: World Cancer Research Fund/American Institute for Cancer Research
