# Supplementary material for: Association between awareness of nutrition labeling and high-density lipoprotein cholesterol concentration in cancer survivors and the general population: The Korean National Health and Nutrition Examination Survey (KNHANES) 2010–2016
Source: BMC Cancer. 2019 Jan 7;19:16. doi: 10.1186/s12885-018-5196-6 (PMC6322264; doi:10.1186/s12885-018-5196-6)

**Additional file 1: Table S1. Associations between subject characteristics and serum HDL-C concentrations in cancer survivors and the general population**

| **Variables** | **Cancer Survivor** | | | **General population** | | | **P-value** |
| --- | --- | --- | --- | --- | --- | --- | --- |
|  | **β** | **SE** | **P-value** | **β** | **SE** | **P-value** |  |
| **Awareness on nutrition labelling** |  |  |  |  |  |  |  |
| Checks nutrition facts and makes labeling-dependent purchase decisions | 52.30 | ±12.39 | 0.5012 | 53.33 | ±12.62 | <.0001 | 0.1112 |
| Checks nutrition facts but does not make labeling-dependent purchase decisions/ Aware of nutrition facts but does not check them when making food purchase decisions | 50.24 | ±12.04 |  | 50.75 | ±12.19 |  |  |
| Unaware of nutrition facts | 49.71 | ±13.98 |  | 47.84 | ±11.77 |  |  |
| **Sex** |  |  |  |  |  |  |  |
| Male | 47.86 | ±12.93 | <.0001 | 46.73 | ±11.32 | <.0001 | 0.4078 |
| Female | 51.91 | ±12.83 |  | 52.86 | ±12.33 |  |  |
| **Age (years)** |  |  |  |  |  |  |  |
| 30-39 | 54.07 | ±11.62 | 0.5175 | 52.60 | ±12.37 | <.0001 | 0.7919 |
| 40-49 | 52.30 | ±13.41 |  | 51.31 | ±12.25 |  |  |
| 50-59 | 52.40 | ±13.56 |  | 50.40 | ±12.38 |  |  |
| 60+ | 48.98 | ±12.68 |  | 47.77 | ±11.76 |  |  |
| **Educational level** |  |  |  |  |  |  |  |
| Under high school graduation | 49.83 | ±12.99 | 0.2773 | 49.60 | ±12.25 | 0.6023 | 0.2053 |
| Bachelor`s degree | 51.99 | ±12.76 |  | 51.67 | ±12.28 |  |  |
| Master`s degree or above | 50.88 | ±14.78 |  | 50.29 | ±12.27 |  |  |
| **Household income** |  |  |  |  |  |  |  |
| Low | 48.50 | ±12.21 | 0.5734 | 47.88 | ±12.04 | <.0001 | 0.9079 |
| Mid-low | 50.27 | ±12.96 |  | 50.04 | ±12.13 |  |  |
| Mid-high | 51.31 | ±13.49 |  | 50.98 | ±12.22 |  |  |
| High | 51.83 | ±13.48 |  | 51.37 | ±12.46 |  |  |
| **BMI** |  |  |  |  |  |  |  |
| <23 | 53.73 | ±13.19 | <.0001 | 53.73 | ±12.71 | <.0001 | 0.1500 |
| 23-25 | 49.16 | ±13.73 |  | 48.97 | ±11.57 |  |  |
| >25 | 45.46 | ±10.17 |  | 46.54 | ±10.86 |  |  |
| **Aerobic exercise habits** |  |  |  |  |  |  |  |
| Yes | 52.04 | ±13.14 | 0.0553 | 51.42 | ±12.69 | <.0001 | 0.4478 |
| No | 49.62 | ±12.91 |  | 49.81 | ±12.10 |  |  |
| **Smoking status** |  |  |  |  |  |  |  |
| Non-smoker | 50.40 | ±12.71 | 0.4958 | 50.98 | ±12.21 | <.0001 | 0.0984 |
| Smoker | 49.53 | ±15.55 |  | 47.29 | ±12.22 |  |  |
| **High risk drinking** |  |  |  |  |  |  |  |
| No | 50.14 | ±12.82 | 0.0249 | 50.12 | ±12.15 | <.0001 | 0.7964 |
| Yes | 53.45 | ±16.18 |  | 51.76 | ±13.46 |  |  |
| **Family history for hyperlipidemia** |  |  |  |  |  |  |  |
| No | 50.25 | ±12.99 | 0.9565 | 50.17 | ±12.25 | 0.3075 | 0.8530 |
| Yes | 52.24 | ±14.15 |  | 52.62 | ±12.95 |  |  |
| **Survey year** |  |  |  |  |  |  |  |
| 2010 | 49.60 | ±12.45 | 0.2518 | 48.42 | ±11.10 | <.0001 | 0.2640 |
| 2011 | 51.40 | ±13.87 |  | 50.39 | ±12.88 |  |  |
| 2012 | 48.23 | ±10.51 |  | 50.13 | ±11.89 |  |  |
| 2013 | 53.11 | ±12.60 |  | 50.76 | ±11.67 |  |  |
| 2014 | 50.96 | ±12.45 |  | 51.04 | ±11.99 |  |  |
| 2015 | 49.24 | ±13.63 |  | 50.48 | ±12.99 |  |  |
| 2016 | 50.27 | ±14.52 |  | 51.29 | ±13.31 |  |  |
| **Stress awareness** |  |  |  |  |  |  |  |
| Low | 50.49 | ±13.33 | 0.4461 | 50.19 | ±12.26 | 0.8449 | 0.3982 |
| High | 49.65 | ±11.81 |  | 50.58 | ±12.40 |  |  |
| **Subjective health status** |  |  |  |  |  |  |  |
| Good | 48.31 | ±12.29 | 0.0622 | 51.19 | ±12.35 | <.0001 | 0.0039 |
| Normal | 51.65 | ±13.21 |  | 50.21 | ±12.28 |  |  |
| Bad | 49.60 | ±13.04 |  | 48.77 | ±12.09 |  |  |
| **The frequency of eating out** |  |  |  |  |  |  |  |
| More than five times a week | 50.81 | ±12.64 | 0.7157 | 49.90 | ±12.02 | 0.9414 | 0.7041 |
| Less than four times a week | 50.21 | ±13.10 |  | 50.47 | ±12.43 |  |  |
| **Total** | 50.31 | ±13.01 |  | 50.29 | ±12.29 |  |  |

| **Variables** | | **Total** | | | | **Cancer Survivor** | | | | | **General population** | | | |
| --- | --- | --- | --- | --- | --- | --- | --- | --- | --- | --- | --- | --- | --- | --- |
|  |  | **RR** | **95% CI** | | **P-value** | **RR** | **95% CI** | | **P-value** | **RR** | | **95% CI** | | **P-value** |
| **Total cholesterol** | Active use | 0.9996 | 0.9985 | 1.0007 | 0.4650 | 1.0048 | 0.9987 | 1.0110 | 0.1259 | 0.9995 | | 0.9984 | 1.0006 | 0.3665 |
|  | Use | 1.0008 | 0.9999 | 1.0016 | 0.0787 | 1.0015 | 0.9971 | 1.0059 | 0.5085 | 1.0007 | | 0.9999 | 1.0016 | 0.0879 |
| **Triglyceride** | Active use | 0.9865 | 0.9759 | 0.9972 | 0.0138 | 0.9791 | 0.9250 | 1.0364 | 0.4669 | 0.9868 | | 0.9760 | 0.9977 | 0.0177 |
|  | Use | 0.9929 | 0.9847 | 1.0013 | 0.0984 | 0.9893 | 0.9486 | 1.0316 | 0.6136 | 0.9932 | | 0.9847 | 1.0017 | 0.1152 |
| **HDL cholesterol** | Active use | 1.0012 | 0.9995 | 1.0030 | 0.1785 | 1.0117 | 1.0001 | 1.0233 | 0.0479 | 1.0009 | | 0.9992 | 1.0027 | 0.3042 |
|  | Use | 1.0017 | 1.0004 | 1.0031 | 0.0131 | 1.0121 | 1.0036 | 1.0205 | 0.0050 | 1.0015 | | 1.0001 | 1.0028 | 0.0349 |
| **LDL cholesterol** | Active use | 0.9998 | 0.9959 | 1.0036 | 0.9051 | 1.0005 | 0.9797 | 1.0217 | 0.9602 | 0.9997 | | 0.9958 | 1.0037 | 0.8894 |
|  | Use | 1.0025 | 0.9995 | 1.0055 | 0.1002 | 1.0052 | 0.9899 | 1.0207 | 0.5087 | 1.0024 | | 0.9994 | 1.0054 | 0.1227 |

**Table S2. Multiple regression analysis of the association between nutrition labeling awareness and outcome variables in cancer survivors and the general population**

*Checks nutrition facts and makes labeling-dependent purchase decisions= Active use, Checks nutrition facts but does not make labeling-dependent purchase decisions/ Aware of nutrition facts but does not check them when making food purchase decisions= Use. Adjusted sex, age, educational level, household income, BMI, aerobic exercise habits, smoking, high risk drinking, family history for hyperlipidemia, year, stress, subjective health, frequency of eating out, total energy intake, daily carbohydrate intake, daily fat intake, total cholesterol, triglyceride, and LDL-C.

**Figure S1.** The results of the subgroup analyses of the relationship between nutritional information awareness and HDL-C levels according to sex, frequency of eating out, and subjective health status. *The RR as marked to the square points was calculated by multiple regression analysis adopting gamma distribution to investigate the association between awareness on nutrition labelling and HDL-C. Results were considered statistically significant if each bar marked to SD did not reach the cut-off line of 1.0000. † The means and SD of each group were shown in supplementary file 3.


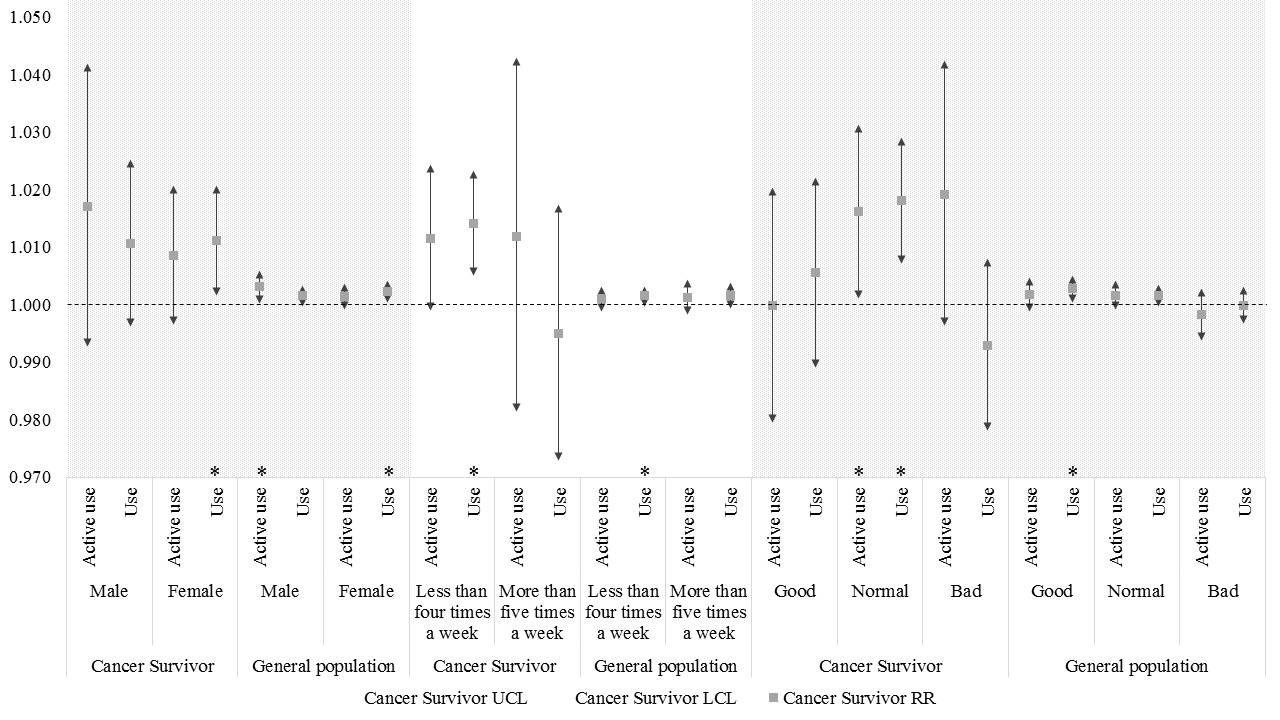

Supplement: Supplementary file 1 — Table S1. Associations between subject characteristics and serum HDL-C concentrations in cancer survivors and the general population. Table S2. Multiple regression analysis of the association between nutrition labeling awareness and outcome variables in cancer. Figure S1. The results of the subgroup analyses of the relationship between nutritional information awareness and HDL-C levels according to sex, frequency of eating out, and subjective health status. *The RR as marked to the square points was calculated by multiple regression analysis adopting gamma distribution to investigate the association between awareness on nutrition labelling and HDL-C. Results were considered statistically significant if each bar marked to SD did not reach the cut-off line of 1.0000. † The means and SD of each group were shown in Additional file 1 Figure S1. (DOCX 251 kb) [file 12885_2018_5196_MOESM1_ESM.docx]
